# Human cytomegalovirus escapes immune recognition by NK cells through the downregulation of B7-H6 by the viral genes US18 and US20

**DOI:** 10.1038/s41598-017-08866-2

**Published:** 2017-08-17

**Authors:** Yoav Charpak-Amikam, Tobias Kubsch, Einat Seidel, Esther Oiknine-Djian, Noemi Cavaletto, Rachel Yamin, Dominik Schmiedel, Dana Wolf, Giorgio Gribaudo, Martin Messerle, Luka Cicin-Sain, Ofer Mandelboim

**Affiliations:** 1 The Lautenberg Center for General and Tumor Immunology, Institute for Medical Research Israel-Canada (IMRIC), Faculty of Medicine, Hebrew University Hadassah Medical School, Jerusalem, 91120 Israel; 2 Department for Vaccinology/Immune Aging and Chronic Infection, HZI, 38124 Braunschweig, Germany; 3 Clinical Virology Unit, Hadassah Hebrew University Medical Center, Jerusalem, 91120 Israel; 4 Department of Life Sciences and Systems Biology, University of Turin, 10123 Turin, Italy; 5 Institute for Virology, Medical School Hannover, 30625 Hannover, Germany; 6 Laboratory of Molecular Genetics and Immunology, The Rockefeller University, 1230 York Avenue, New York, NY 10065 USA

## Abstract

Human cytomegalovirus (HCMV) is a major human pathogen, causing serious diseases in immunocompromised populations and congenially infected neonates. One of the main immune cells acting against the virus are Natural Killer (NK) cells. Killing by NK cells is mediated by a small family of activating receptors such as NKp30 that interact with the cellular ligand B7-H6. The outcome of B7-H6-NKp30 interaction was, so far, mainly studied with regard to NK recognition and killing of tumors. Here, we demonstrated that the expression of B7-H6 is upregulated following HCMV infection and that HCMV uses two of its genes: US18 and US20, to interfere with B7-H6 surface expression, in a mechanism involving endosomal degradation, in order to evade NK cell recognition.

## Introduction

HCMV, also known as Human Herpesvirus 5 (HHV-5), is an important human pathogen, a member of the Betaherpesvirus family. It infects the majority of the human population, and following primary infection, it can persists as a life-long infection^1^. While healthy individuals experience the infection as mild or sub-clinical, HCMV poses a major threat to immune-compromised populations such as transplant recipients or AIDS patients, and is a significant cause of infection-related congenital defects and abortions^2, 3^. The virus contains a dsDNA genome, the largest of the herpesvirus family, which codes for hundreds of genes. Out of these, many are used to evade immune recognition^4–7^, with an emphasis on escape from the innate immune attack^8, 9^.

NK cells that belong to the innate immunity system play a critical role in fighting HCMV infections. Indeed, individuals that suffer from NK cell deficiency suffer from a higher susceptibility to different herpesvirus infections, including HCMV^10^. NK cells have recently also been classified as a cytotoxic type of Innate Lymphoid Cells (ILCs)^11^. They constitute 5–15% of the lymphocytes in healthy peripheral blood, and are capable of killing virally-infected cells, tumor cells^12^, bacteria^13, 14^ and fungi^15^. NK cells function through the secretion of inflammatory cytokines such as IFNγ, and through direct lysis of cells^12^. The decision of whether or not to kill an inspected cell is mediated by a balance of signals generated by inhibitory and activating receptors. Inhibitory receptors mainly recognize self-molecules such as MHC class I and PVR^12, 16^ Activating receptors recognize several ligands which can be either self or non-self (pathogen-derived) and are upregulated following cellular stress, cancerous process or infection^17^. NKG2D is an NK killer receptor that recognizes 8 human stress induced ligands: MICA, MICB and ULBP 1–6^18^. MICA has dozens of different alleles^19^ that include long cytoplasmic tail proteins and short tail proteins such as MICA *008 that is GPI-linked to the membrane^20^. The expression of NKG2D ligands, including the long and short alleles of MICA, is prevented during HCMV infection by both protein and microRNA-based mechanisms^8^.

NKp30 is a major NK activating receptor. Known ligands of NKp30 include the *Plasmodium falciparum* protein PfEMP-1^21^, the cellular nuclear factor BAT3^22, 23^, and the cellular membrane protein B7-H6^24^, while recognition of the HCMV protein pp65 is inhibitory to NKp30-mediated killing^25^.

B7-H6 contains two Ig-like domains, and its structure bound to NKp30 has been solved^26^. While it is not found to be expressed on healthy cells, it has been shown to be upregulated on the surface of both solid and hematologic transformed cells^24^. The exact mechanisms controlling B7-H6 expression are still largely unknown, but it was demonstrated that several TLR ligands and pro-inflammatory cytokines can induce its expression in non-transformed cells^27^.

The role played by B7-H6 in tumor surveillance has been quite extensively studied. However, its function in anti-viral immunity remains poorly explored. Only recently, B7-H6 was shown to be involved in viral infection, as it was demonstrated that B7-H6 is downregulated during infection with another member of the Betaherpesvirus family, HHV-6^28^. The viral protein responsible for this downregulation is unknown.

Here we show that HCMV, via US18 and US20, downregulates B7-H6 surface expression during infection to escape NK cell attack.

## Results

### The HCMV US14-22 genomic region encodes several immune evasion mechanisms

The HCMV genome contains many immune evasion genes, several of which are designed to prevent NK cell recognition of infected cells. One gene family coding for such proteins is the US12 gene family that includes a set of ten contiguous tandemly arranged genes (US12 to US21) in the unique short (US) region of the HCMV genome^4–7^. Among the US12 genes, US18 and US20 were previously shown to downregulate the NKG2D ligand MICA^29^.

**Figure 1:**
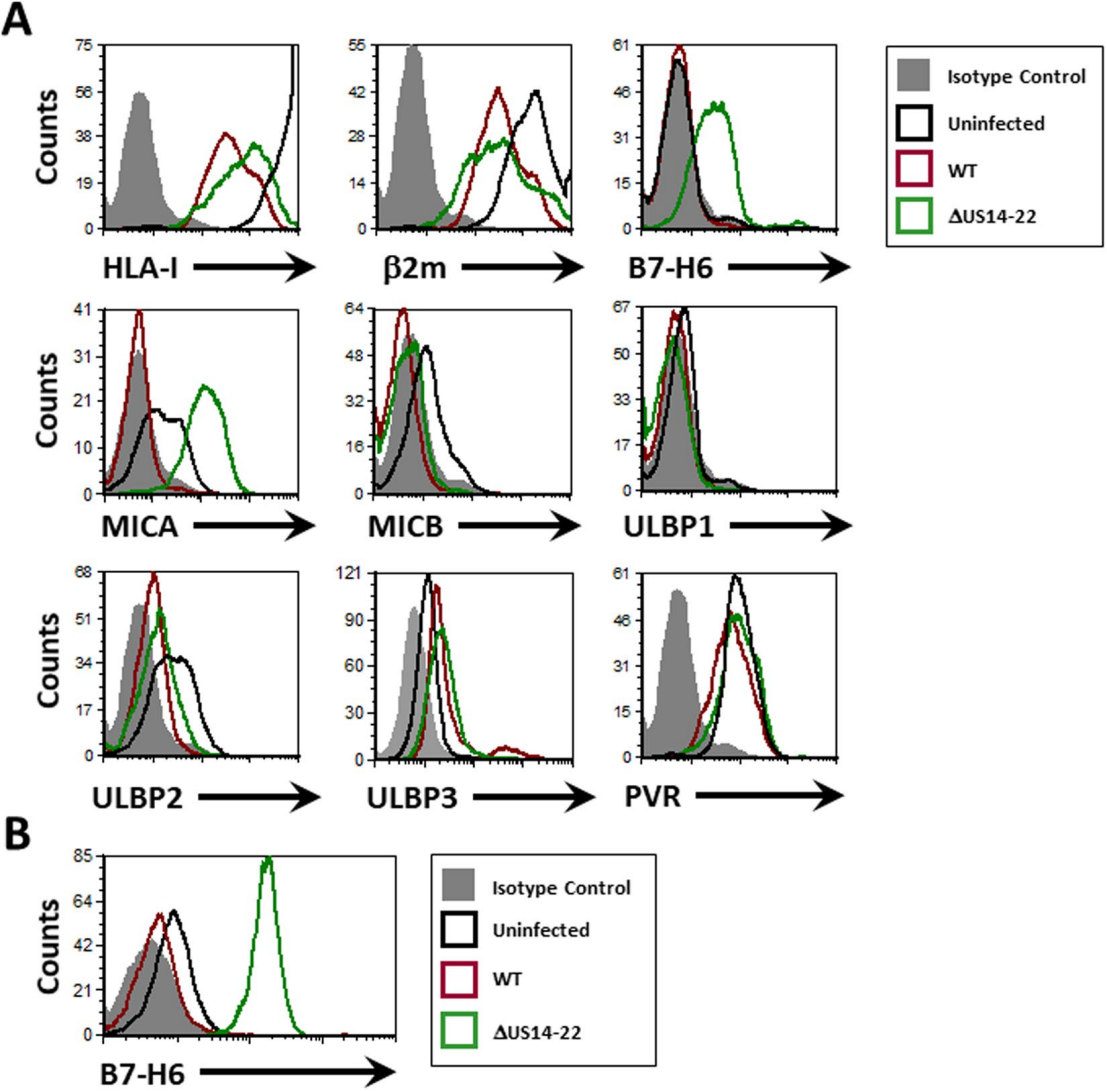
The HCMV US14-22 genomic region encodes several immune evasion mechanisms. (**A**) FACS staining for different NK cell ligands on Human Foreskin Fibroblast cells, 96 hours after infection with WT HCMV TB40/e or HCMV TB40/e deleted for the US14-22 genomic region (or control uninfected cells, as depicted in the figure legend). Filled gray histograms represent staining with the relevant isotype control antibodies. A representative isotype control staining derived from the uninfected cells is presented as no significant differences were observed between isotype control stainings for each infection. (**B**) FACS staining of B7-H6 surface expression on ARPE19 cells infected with different HCMV strains as depicted in the figure legend. No significant differences were observed between isotype control stainings of each infection, so a representative isotype control staining derived from the uninfected cells is presented.

In order to identify additional NK evasion mechanisms mediated by the US12 gene family, we infected primary Human Foreskin Fibroblast (HFF) cells with two strains of HCMV: a wild type TB40/e virus and a TB40/e mutant virus deleted for the US14-22 genomic region (ΔUS14-22). 96 hours following infection, the cells were stained for expression of various NK ligands. As previously described^30^ both HCMV strains reduced surface expression of HLA-I, β2-microglobulin (β2 m), MICB and ULBP2, the last two being ligands for the activating receptor NKG2D^18^ (Fig. 1A). No significant change in ULBP1 or PVR surface expression was detected and a slight increase in ULBP3 levels during infection with both strains was noticed (Fig. 1A).

An increase in MICA surface expression was observed after infection with HCMV ΔUS14-22, while infection with the WT strain led to a complete repression of MICA surface expression (Fig. 1A). This is because the HFF line used here (FLS1) express two full-length MICA alleles that are targeted by US18 and US20 that are present in the US12 region^29, 31^. Interestingly, an increase in B7-H6 surface expression levels was detected, but only during infection with the HCMV strain lacking the US14-22 genomic region (Fig. 1A). To further corroborate these findings we infected the human retinal pigment epithelium cell line ARPE19, and observed a very pronounced upregulation of B7-H6 in the US14-22-deleted virus (Fig. 1B). Since B7-H6 is already expressed on the surface of ARPE19 cells (as opposed to the HFF negative cells, Fig. 1A), we also observed that the WT TB40/e strain was able to downregulate the endogenous B7-H6 expression (Fig. 1B).

### HCMV US18 and US20 are responsible for the B7-H6 downregulation

**Figure 2:**
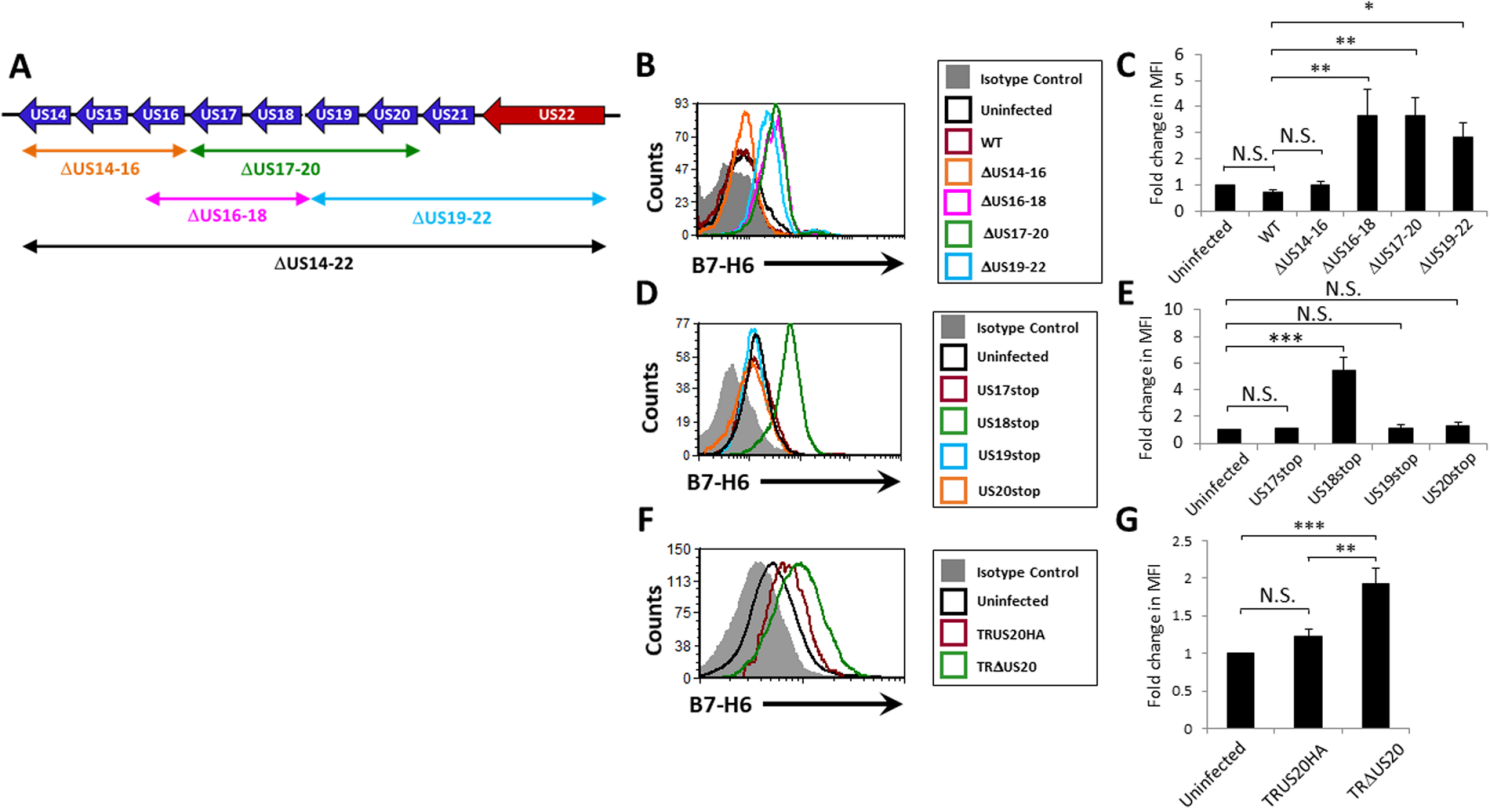
HCMV US18 and US20 are responsible for the B7-H6 downregulation phenotype. (**A**) Schematic representation of HCMV US14-US22 genomic region and the different block deletion mutants used in this study. Blue arrows represent US12-family genes. Red arrows represent US22-family genes. Double headed arrows represent genomic regions deleted in the different block-deletion mutants. (**B**) FACS staining of B7-H6 surface expression on ARPE19 cells infected with different HCMV TB40/e strains deleted for gene blocks within the US14-22 genomic region. Histogram colors are as depicted in the figure legend. One representative experiment out of seven is presented. No significant differences were observed between isotype control stainings of each infection, so a representative isotype control staining derived from the uninfected cells is presented. (**C**) Quantification of the results presented in B. Averages of seven experiments are presented. Error bars represent standard error of the mean. (**D**) FACS staining of B7-H6 surface expression on ARPE19 cells infected with different HCMV TB40/e strains in which the start codon of one ORF out of the US17-20 region was replaced with a stop codon. Histogram colors are as depicted in the figure legend. One representative experiment out of three is presented. No significant differences were observed between isotype control stainings of each infection, so a representative isotype control staining derived from the uninfected cells is presented. (**E**) Quantification of the results presented in D. Averages of three experiments are presented. Error bars represent standard error of the mean. (**F**) FACS staining of B7-H6 surface expression on ARPE19 cells infected with different HCMV TR derivatives. Histogram colors are as depicted in the figure legend. One representative experiment out of four is presented. No significant differences were observed between isotype control stainings of each infection, so a representative isotype control staining derived from the uninfected cells is presented. (**G**) Quantification of the results presented in F. Averages of four experiments are presented. Error bars represent standard error of the mean. ANOVA tests were performed in order to evaluate significance. *p-value < 0.05. **p-value < 0.005. ***p-value < 0.0005. N.S. = non-significant differences.

Next, we wanted to determine whether one or more of the canonical genes encoded within the US14-22 genomic region is responsible for B7-H6 downregulation. We deleted overlapping groups of genes within this region: US14-16, US16-18, US17-20 and US19-22 (schematically described in Fig. 2A), by replacing them with a kanamycin resistance gene. ARPE19 cells were infected with these mutant viruses and B7-H6 expression was determined using flow cytometry. While HCMV deleted for US14-16 retained the same phenotype as the WT virus, deletion of either US16-18, US17-20 or US19-22 resulted in loss of ability to downregulate B7-H6 (Fig. 2B, quantified in Fig. 2C). The fact that deletion of two non-overlapping regions (US16-18 and US19-22) interfered with the virus’ ability to prevent B7-H6 surface expression indicates the existence of at least two non-redundant genetic elements within these two genomic regions that are required for the observed effect. Because similar observations were made with ΔUS17-20 which partially overlaps both suspected regions, we hypothesized that the two genes might be US18 and US20.

To identify the specific genes involved, we generated single deletions in the US17-20 region. The expression of HCMV genes US18, US19 and US20 is dictated by a complex regulatory mechanism that is not yet fully understood. Under different conditions and at different infection time points US18, US19 and US20 can be transcribed as a tri-cistronic mRNA containing all three ORFs^32^. However, US18 can also be transcribed as an independent transcript, and it is possible that a US19-20 transcript also exists^32^. To avoid the possibility that complete deletion of one of these ORFs will lead to interference with the transcription of other genes included in this poly-cistronic mRNA, we chose to interfere with the expression of the suspected genes only at the level of translation, by replacing the start codon of each of them with a stop codon (ATG→TAA). Thus, we generated four silent deletion strains: US17stop, US18stop, US19stop and US20stop. ARPE19 cells were infected with these strains and B7-H6 surface expression levels were again examined using FACS. Only HCMV US18stop was unable to inhibit B7-H6 surface expression (Fig. 2D quantified in Fig. 2E).

This result was surprising because we inferred from our block deletion mutants that both US18 and US20 might be involved in the B7-H6 downregulation. Furthermore, HCMV US18 and US20 were previously shown to act together in the downregulation of MICA^29^. We therefore wondered whether indeed US20 is not involved in the B7-H6 downregulation.

US20 contains a second ATG start codon, in frame, 39 nucleotides downstream of the first start codon that we have replaced. We therefore speculated that it is possible that the virus is able to translate a truncated but still functional US20 protein using this start codon, in our US20stop HCMV mutants. Alternatively, it is possible that US20 is transcribed from a non-canonical start codon that were previously shown to be prevalent in the HCMV genome^6^. Thus, to test whether indeed US20 is not involved in the B7-H6 downregulation, we infected ARPE19 cells with HCMV TRΔUS20 strain, a derivative of the clinical isolate strain TR, in which the entire US20 ORF was replaced with a *galK-Kan* cassette^33^. As a control, we used the TRUS20HA revertant virus, in which a HA-tagged US20 ORF was reconstituted into the deleted region of TRΔUS20^33^. We observed that cells infected with HCMV TRΔUS20 expressed two-fold higher levels of surface B7-H6, which was not observed with the revertant virus (Fig. 2F, quantified in Fig. 2G). Consequently, we concluded that the HCMV genes US18 and US20 act in a non-redundant fashion to downregulate the expression of the NK activating ligand B7-H6 from the surface of infected cells.

### Exogenous overexpression of US18 and US20 is sufficient for reducing B7-H6 surface expression

**Figure 3:**
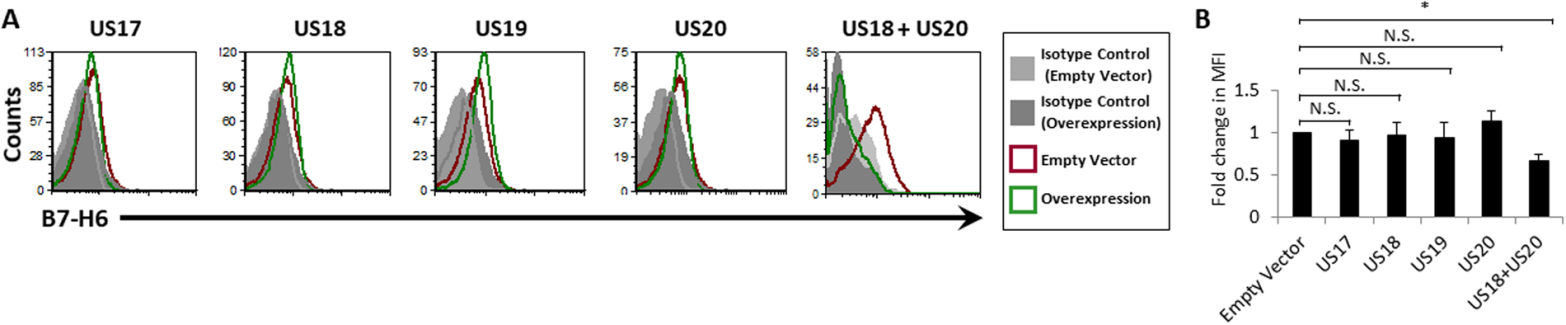
Exogenous overexpression of US18 and US20 is sufficient for reducing B7-H6 surface expression. (**A**) FACS staining for B7-H6 on ARPE19 cells overexpressing HCMV US17, US18, US19 or US20, as presented above the relevant graphs. Histogram colors are as depicted in the figure legend. One representative experiment out of ten is presented. (**B**) Quantification of the results presented in A. Averages of four to ten experiments are presented. Error bars in B represent standard error of the mean. An ANOVA test was performed in order to evaluate significance. *p-value < 0.05. N.S. = non-significant differences.

Our previous set of experiments using HCMV deletion mutants has demonstrated that US18 and US20 are non-redundant in their capacity to downregulate B7-H6. To determine whether they are sufficient or whether they work in concert with additional HCMV genes, we overexpressed 6XHis-tagged US17, US18, US19 or US20 in ARPE19 cells by using lentivirus-based vectors. No difference in B7-H6 surface expression was observed between cells overexpressing the different proteins and cells carrying the empty vector alone (Fig. 3A). We next performed another round of lentivirus transduction, this time overexpressing HA-tagged US20 on the background of US18-His, and identified a significant (p = 0.0077) reduction in B7-H6 levels (Fig. 3A, quantified in Fig. 3B). Thus, only US18 and US20 together are sufficient for B7-H6 downregulation.

### B7-H6 is sent to degradation during HCMV infection

**Figure 4:**
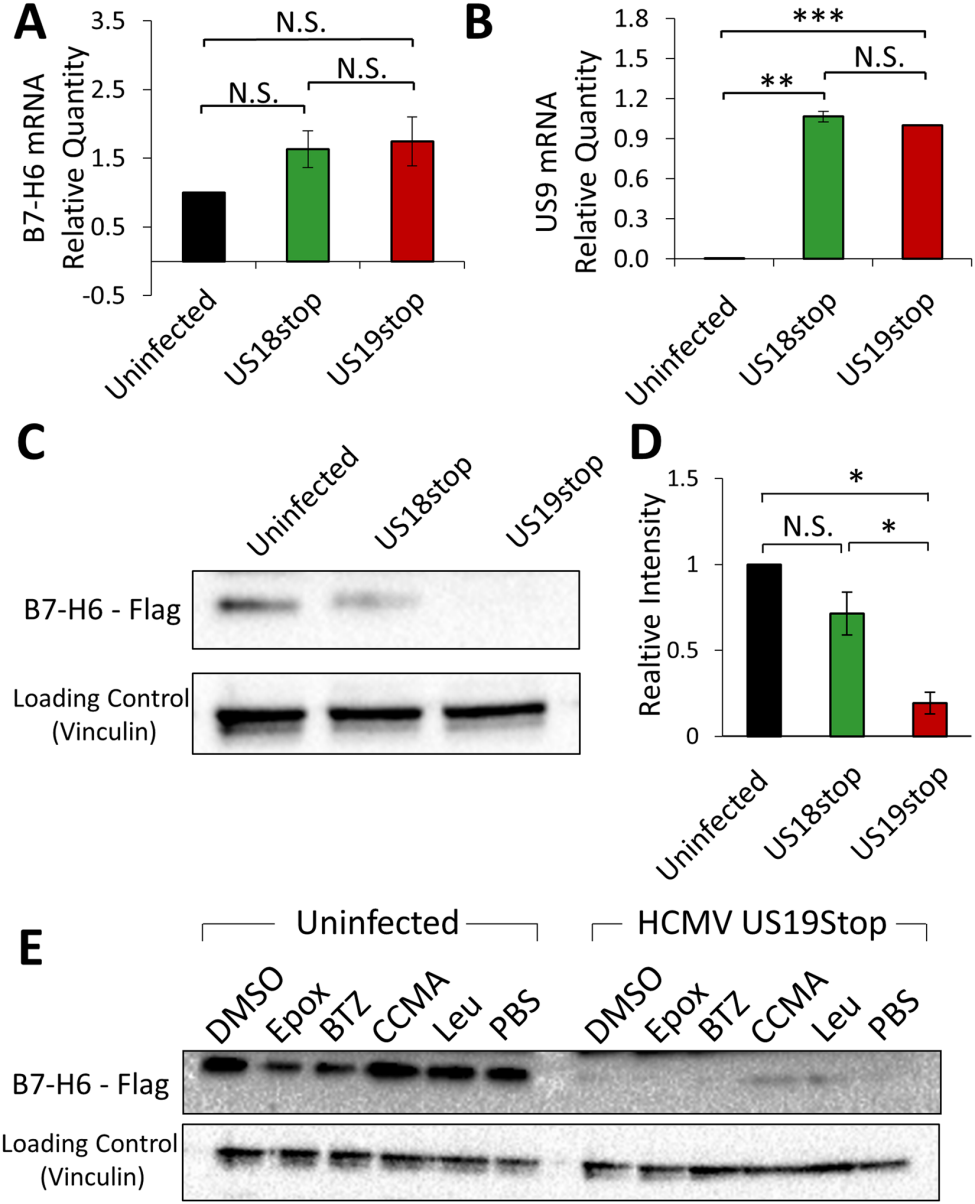
HCMV sends B7-H6 to degradation. (**A**) Quantitative RT-PCR for B7-H6 expression in ARPE19 cells infected with HCMV US18stop, HCMV US19stop or left uninfected. Bar identities are as depicted in the figure. Relative B7-H6 mRNA quantity was normalized to the uninfected cells samples. The presented results are averages of 3 biological repeats. Error bars represent standard error of the mean. (**B**) Quantitative real-time PCR for US9 expression in ARPE19 cells infected with HCMV US18stop, HCMV US19stop or left uninfected. Bar identities are as depicted in the figure. Relative US9 mRNA quantity was normalized to the samples of cells infected with HCMV US19Stop. The presented results are averages of 3 biological repeats. Error bars represent standard error of the mean. (**C**) Western blot of HCMV infected ARPE19 cells overexpressing FLAG-tagged B7-H6. The upper row presents a blot with an anti-FLAG antibody. The lower row presents a blot with anti-human Vinculin, used as a loading control. All samples derived from the same experiment were processed in parallel. One representative experiment out of three is presented. (**D**) Quantification of the results presented in C. Averages of three independent experiments are presented. Error bars represent standard deviation. (**E**) Western blot of HCMV infected ARPE19 cells overexpressing FLAG-tagged B7-H6 and treated with DMSO (10 μl/ml), Epoxomycin (Epox, 10 μM), Bortezomib (BTZ, 500 μM), Concanamycin A (CCMA, 50 nM), Leupeptin (Leu, 500 μM) or left untreated (PBS). The upper row presents a blot with an anti-FLAG antibody. The lower row presents a blot with anti-human Vinculin, used as a loading control. The presented blots were cropped and the background was adjusted for clarity. A student’s t test was performed in order to evaluate significance. *p-value < 0.05. **p-value < 0.005. ***p-value < 0.0005. N.S. = non-significant differences.

To identify the molecular mechanism by which US18 and US20 inhibit B7-H6 surface expression we initially determined the B7-H6 mRNA levels. We extracted mRNA from uninfected ARPE19 cells, and ARPE19 cells infected with either the mutant US18stop that is unable to reduce B7-H6 surface expression, or with the US19stop mutant which retains the ability to downregulate B7-H6 and observed no change in mRNA levels of B7-H6 (Fig. 4A). Infection levels of US18stop and US19stop were similar as determined by qRT-PCR experiments using the HCMV gene US9 (Fig. 4B).

Next, we tested whether the decrease in B7-H6 surface expression is due to a general decrease in cellular B7-H6 protein quantity, as has been shown for the downregulation of MICA by US18 and US20^29^. Since the expression of B7-H6 in ARPE19 cells is low, we stably overexpressed B7-H6 fused to a C-terminal FLAG-tag in these cells. FACS experiments were then performed in order to validate the increased B7-H6 expression and the ability of HCMV US18stop and US19stop to replicate the effects observed for endogenous B7-H6 (Sup. Fig. S1). We then infected the cells with HCMV US18Stop or US19Stop, and 72 hours post-infection prepared protein lysates. Western blot experiments were performed using antibodies against the FLAG-tag (as the commercial anti-B7-H6 mAb does not work in western blot assays), while Vinculin levels were used as a loading control. Interestingly, a strong decrease in B7-H6 protein levels was observed during infection with US19stop, (Fig. 4C, quantified in 4D), while infection with US18Stop had no effect, suggesting that during infection B7-H6 is being degraded.

### Lysosomal degradation is involved in the HCMV-induced B7-H6 downregulation

To further study the mechanism by which HCMV degrades B7-H6 we repeated the western blot experiment depicted in Fig. 4C in the presence of two proteasome inhibitors – Epoxomycin (Epox) and Bortezomib (BTZ), and two lysosome inhibitors – Concanamycin A (CCMA) and Leupeptin (Leu). While proteasome inhibition didn’t rescue B7-H6 levels during infection, an increase in the protein levels was detected when lysosomal inhibitors were used (Fig. 4E).

**Figure 5:**
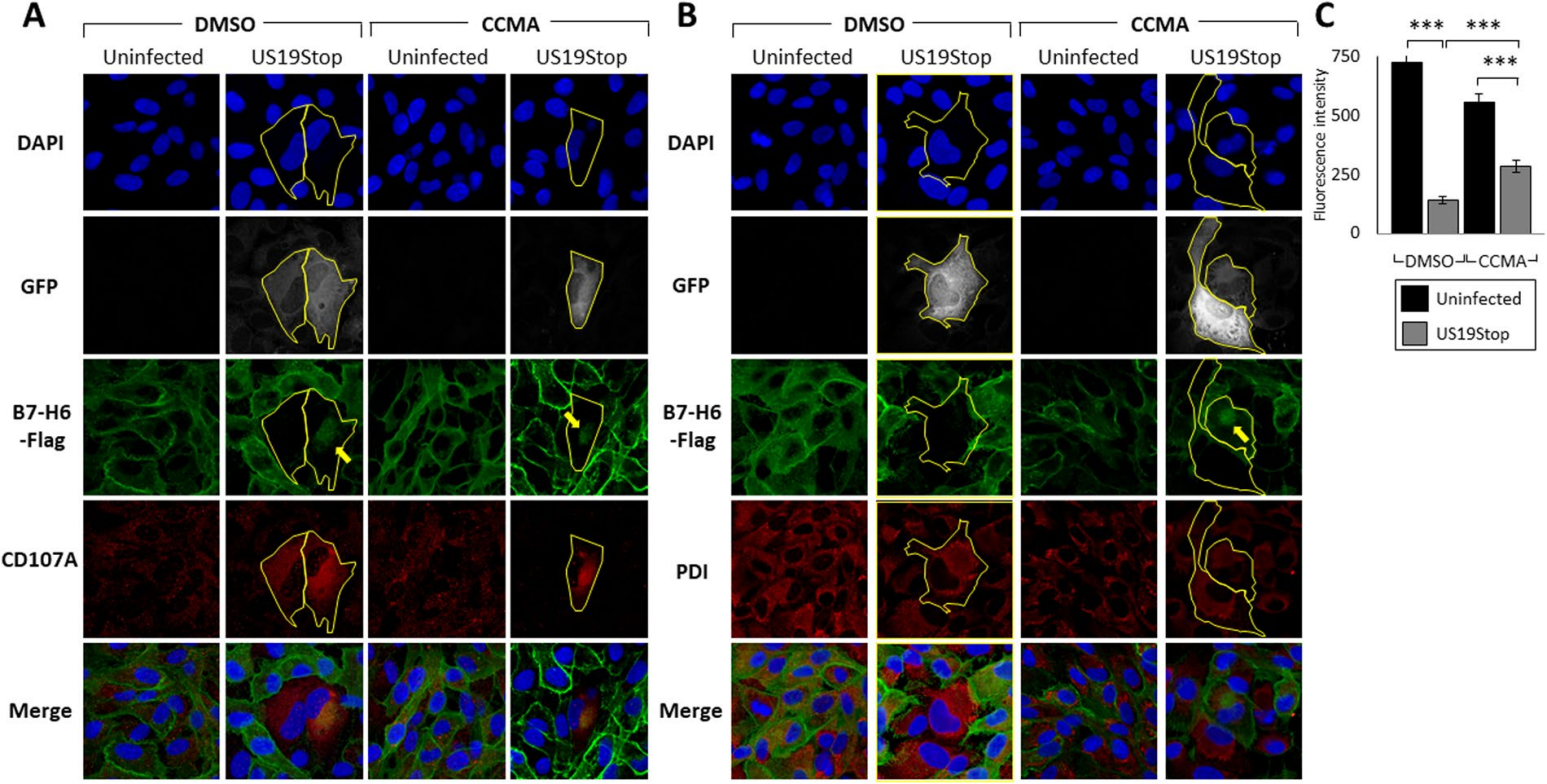
Downregulation of B7-H6 during HCMV infection is mediated through lysosomal degradation. (**A**) ARPE19 cells overexpressing B7-H6-FLAG were infected with HCMV US19Stop or left uninfected, and were treated with 50 nM Concanamycin A (CCMA) or left untreated (DMSO). The cells were then stained with DAPI and antibodies against the FLAG-tag or the lysosomal marker CD107A, and visualized using confocal microscopy. GFP marks infected cells. Infected cells boundaries are marked by a yellow line. Yellow arrows mark intracellular accumulations of B7-H6-FLAG. (**B**) ARPE19 cells overexpressing B7-H6-FLAG were infected with HCMV US19Stop or left uninfected, and were treated with 50 nM Concanamycin A (CCMA) or left untreated (DMSO). The cells were then stained with DAPI and antibodies against the FLAG-tag or the ER marker PDI, and visualized using confocal microscopy. GFP marks infected cells. Infected cells boundaries are delineated using a yellow line. Yellow arrows mark intracellular accumulations of B7-H6-FLAG. (**C**) Quantification of the FLAG-tag fluorescence intensity shown in A. Averages of 45–85 cells from each category are presented, error bars represent standard error of the mean. A student’s t test was performed in order to evaluate significance. ***p-value < 0.0005.

To further corroborate these data ARPE19 cells stably overexpressing FLAG-tagged B7-H6 were infected for 72 hours with the GFP-expressing HCMV mutant US19Stop or left uninfected, in the presence or absence of the lysosome inhibitor CCMA. The cells were stained for the FLAG-tag (indicative of B7-H6 expression), the nucleus (using DAPI), and for either the lysosomal marker CD107A (Fig. 5A) or the ER marker Protein-Disulfide-Isomerase (PDI) (Fig. 5B). A significant negative correlation was observed between infection levels (determined by GFP) and cellular B7-H6-FLAG levels (Pearson’s coefficient of R = −0.54), indicating that B7-H6 is degraded.

As expected, B7-H6-FLAG was expressed mainly on the cell surface of uninfected cells while in HCMV-infected cells we observed substantially reduced levels of B7-H6-FLAG (Fig. 5A–C, marked by yellow lines). In infected cells treated with the lysosomal inhibitor CCMA, more B7-H6-FLAG was observed and the degree of B7-H6-FLAG degradation was less pronounced (Fig. 5A–C). In addition, under lysosomal inhibition we observed an intracellular, peri-nuclear accumulation of B7-H6-FLAG (Fig. 5A and B, marked by an arrow). This population was seen following infection and increased following the addition of CCMA. We also observed that following HCMV infection, CD107A (a lysosomal marker) was co-localized to the peri-nuclear compartment, together with B7-H6-FLAG, when the lysosome is inhibited (Fig. 5A, CCMA). As previously shown, this peri-nuclear CD107A-positive compartment is most likely the Viral Assembly Compartment^34, 35^. The ER staining (detected by PDI) was not changed during infection and was not co-localized with B7-H6-FLAG (Fig. 5B).

Overall we suggest that during HCMV infection B7-H6 is localized to the Viral Assembly Compartment and later degraded, at least partially, by the lysosomes.

### HCMV downregulation of B7-H6 leads to evasion from NKp30-medited killing by NK cells

**Figure 6:**
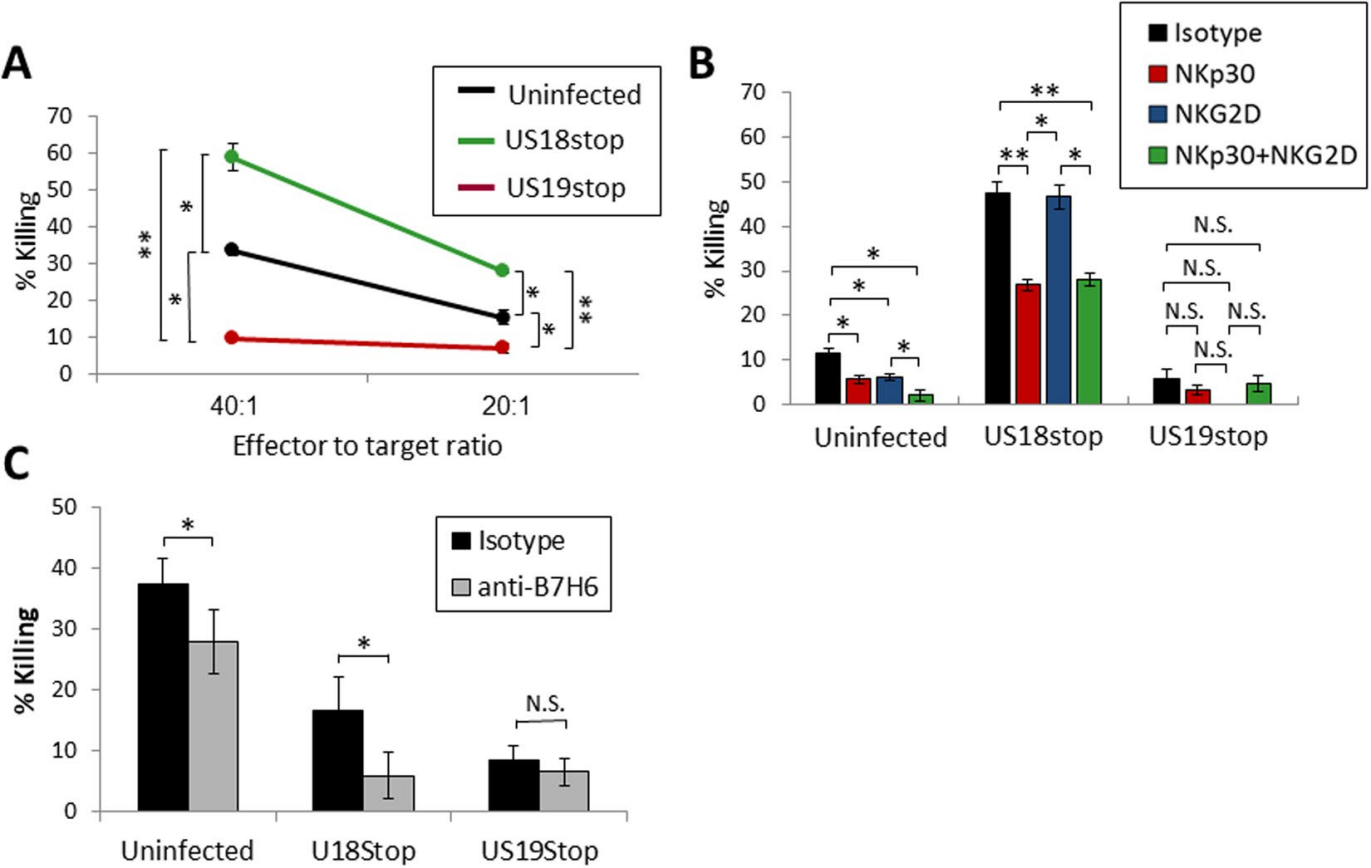
HCMV downregulation of B7-H6 leads to evasion from NKp30-mediated killing by NK cells. (**A**) NK cell killing of ARPE19 cells infected with different HCMV strains as depicted in the figure legend. Two effector to target ratios are presented as depicted on the X-axis. One representative experiment out of four is presented. (**B**) NK cell killing of ARPE19 cells infected with different HCMV strains and blocked using different antibodies as depicted in the figure legend. The isotype control antibody used was an anti-human CD99 antibody. Blocking experiments were performed at a 20:1 effector to target ratio. One representative experiment out of four is presented. (**C**) NK cell killing of ARPE19 cells infected with different HCMV strains and blocked with either isotype control antibody (black bars, polyclonal mouse IgG1) or anti-B7-H6 antibody (grey bars). One representative experiment out of two is presented. For all presented experiments error bars represent standard error of the mean. A student’s t test was performed in order to evaluate significance. *p-value < 0.05. **p-value < 0.005. N.S. = non-significant differences.

Finally, we studied the functional significance of B7-H6 downregulation by US18 and US20 with regard to evasion from NK cells. We incubated ^35^S-Methionine-labeled ARPE19 cells that were left uninfected or infected with HCMV US18stop or with US19stop with primary NK cells and performed NK cytotoxicity assays. Infection with HCMV US19stop led to evasion from NK cell mediated killing, as the infected cells were hardly killed (Fig. 6A).We think that the reduced killing of US19Stop-infected cells is because HCMV uses many mechanisms to evade recognition by NK cells^8^, including the US18 and US20-mediated downregulation of B7-H6 that we show here. In contrast, infection with the US18stop HCMV mutant led to the opposite effect, and considerably increased killing of the infected cells was observed (Fig. 6A). This is probably because of the upregulation in B7-H6 expression observed following HCMV infection in the absence of US18 (see above figures).

To determine the involvement of NKp30, NKG2D or both receptors we repeated the NK killing experiments, this time incubating the NK cells with specific antibodies that block NKp30 and NKG2D interactions. In uninfected cells, a slight reduction in killing was observed following blocking with anti-NKp30, anti-NKG2D or both (Fig. 6B). This reduction was not observed in cells infected with the US19stop virus, probably because of the reduction in the NKG2D and NKp30 ligands. A very pronounced increase in the killing of cells infected with HCMV US18stop was again observed (Fig. 6B), most likely due to the interference with the immune evasion effects of US18 and the increase in B7-H6. We observed a reduction in killing only following blocking of the NKp30 receptor, but not NKG2D (Fig. 6B). Blocking of both receptors resulted in a similar effect as blocking of NKp30 alone (Fig. 6B). Even in the presence of NKp30 inhibition, the reduction in NK killing did not reach the levels observed in uninfected cells or cells infected with US19stop (Fig. 6B), indicating the US18 and probably also US20 are involved in the downregulation of additional NK ligands that are recognized by other activating receptors.

To further corroborate these results we repeated the NK cytotoxicity experiments in the presence of a B7-H6 blocking antibody. While B7-H6 blockage did not significantly affect killing of HCMV U19Stop-infected cells (because B7-H6 was downregulated), it successfully decreased NK cytotoxicity against uninfected and HCMV US18Stop-infected cells (Fig. 6C).

## Discussion

During the course of evolution and the arms race between HCMV and its human host, the virus has developed a large array of immune-evasion mechanisms, many of which are targeted against NK cell recognition of HCMV infected cells.

Here we demonstrate that B7-H6 surface expression is upregulated during HCMV infection, and identify the viral genes that counter this effect. Using a series of HCMV mutants, we determine that the US12-family genes US18 and US20 are necessary for the downregulation of B7-H6 in two clinical strains of HCMV: TB40/e and TR. By overexpressing relevant US12-family member genes in uninfected cells, we show that both US18 and US20 are necessary and sufficient for the downregulation of B7-H6. While no significant change in the levels of B7-H6 mRNA was observed during infection, HCMV mutants lacking US18 were unable to downregulate B7-H6 protein levels. This suggests that the mechanism/s HCMV uses to oppose B7-H6 mediated recognition work through the degradation of B7-H6, similarly to the action of US18 and US20 on the NK activating ligand MICA^29^. Although we cannot completely rule out the possibility that some of the observed decrease in B7-H6 results from shedding of the protein by infected cells, using a series of proteasome and lysosome inhibitors we observed that lysosomal degradation is involved in HCMV degradation of B7-H6 and that prior to its degradation B7-H6 is most likely located in the Viral Assembly Compartment. Finally, we showed that the US18- and US20-mediated downregulation of B7-H6 has a major effect on the ability of HCMV to evade elimination by NK cells.

US18 and US20 are now known to interfere with two NK ligands: MICA - a NKG2D ligand, and B7-H6 - a NKp30 ligand. Our killing data suggest that US18 and US20 probably interfere with the activity of additional NK cell ligands, making these two viral proteins prominent in manipulating NK cell activity.

We previously showed that HCMV directly interferes with NKp30 function through inhibitory binding of the viral protein pp65 to this receptor^25^, and we now show that HCMV also downregulates its ligand. The fact that HCMV manipulates multiple steps in the NKp30 pathway further demonstrates its importance for this particular virus.

Furthermore, we recently demonstrated that another herpesvirus, HHV-6, downregulates B7-H6 through an unknown mechanism^28^. Thus, we speculate that B7-H6 might have a role in the immune escape of additional viruses from other families as well, and that perhaps additional B7-H6 immune evasion mechanisms will also be discovered in HCMV.

The molecular mechanism responsible for B7-H6 induction and downregulation following virus and tumor transformation are still largely unknown. We don’t know what are the cellular signaling and transcription networks leading to the induction of B7-H6 during infection, and whether they differ from the signals used to upregulate B7-H6 during cancerous processes. Answers to these questions might allow us to develop methods to induce B7-H6 and NK-mediated killing during malignant and viral diseases, that otherwise evade such immune recognition and elimination.

While we were preparing this manuscript, another group has conducted a study regarding immune evasion mechanisms of HCMV US12 family genes. The study was performed on the Merlin HCMV strain, whereas in our study we used the TB40/e and TR strains. Importantly, both studies arrive at similar conclusions and thus they complement and strengthen each other, arguing that B7-H6 downregulation by the concerted action of US18 and US20 is a conserved function in several clinical HCMV strains^36^.

In conclusion, we show here that B7-H6 is not only a tumor cell marker, but might be better classified as a member of the stress ligand family of NK ligands, induced following cancer transformation and viral infection, as a method for marking cells for NK-mediated elimination.

## Methods

### Cells, antibodies and reagents

ARPE19 cells (ATCC CRL-2302) and HEK293T cells (ATCC CRL-3216) were obtained from the ATCC. NK cells were cultured from blood donations of healthy adults and isolated and activated as previously described^37^. FLS1 Human foreskin fibroblasts were obtained and cultured from a healthy donor as previously described^31^. All cells originating from primary human donations were obtained in accordance with the institutional guidelines and permissions for using human tissues and informed consent was obtained from all donors. The relevant institutional committees approved all relevant experimental protocols. The relevant institutional Helsinki request number is 0030-12-HMO.

The following primary antibodies were used for Flow cytometry experiments: anti-B7H6 (R&D systems, clone 875001), anti-HLA1 (W6/32), anti-beta2m (clone -02), anti-MICA (R&D systems, clone 159227), anti-MICB (R&D systems, clone 236511), anti-ULBP1 (R&D systems, clone 170818), anti-ULBP2 (R&D systems, clone 165903), anti-ULBP3 (R&D systems, clone 166510), anti-PVR (in-house developed), mouse IgG1 isotype control (Biolegend, clone MOPC-21), mouse IgG2a isotype control (Biolegend, clone MOPC-173), and mouse IgG2b isotype control (Biolegend, clone MPC-11).

The following primary antibodies were used for western blot experiments: anti-FLAG (Biolegend, clone L5) and anti-Vinculin (Abcam EPR8185).

The following Antibodies were used for blocking of receptors or ligands during NK cytotoxicity experiments: anti-NKp30 (Biolegend, clone p30-15), anti-NKG2D (R&D systems, clone 149810) anti-CD99 (clone 12E7, a gift from A. Bernard, INSERM, France) and anti-B7-H6 (R&D systems, clone 875001).

The following secondary antibodies were used in relevant experiments: anti-mouse-AlexaFluor-647, anti-rat-HRP, anti-rabbit-HRP, anti-rat-AlexaFluor-647 and anti-rabbit-Cy3, all purchased from Jackson laboratories. For immunofluorescence microscopy experiments the following primary antibodies were used: rat anti-FLAG (BioLegend, clone L5), rabbit anti-CD107A (AB2971, Merck Millipore), rabbit anti-PDI (Ab3672, Abcam).

For proteasome inhibition the following reagents were used: Epoxomycin (A2606, Apexbio) and Bortezomib (BNB-1408, LC Biolabs). For lysosomal inhibition Concanamycin A (27689, Sigma) and Leupeptin (Merck Millipore, 108976) were used.

### Gene cloning and overexpression in cells

Lentiviral vectors containing different genes for overexpression were constructed using the TransIt-LT1 transfection reagent (Mirus 2306) and a transient three-plasmid protocol as previously described^38^. In brief, the human gene B7-H6 and the HCMV genes US17, US18, US19 and US20 were amplified from cDNA prepared from RNA of ARPE19 cells infected with HCMV TB40/e as described in the quantitative real-time PCR protocol in the methods section. The genes were amplified using primers containing the relevant tags as detailed in Sup. Table S1. The amplified genes were inserted into one of two plasmid vectors - pHAGE-DsRED(−)-eGFP(+) or SIN18-pRLL-hEFIap-E-GFP-WRPE, that were used to prepare lentiviruses in HEK293T cells. The lentiviruses were used to transduce cells and overexpress relevant proteins. Transduction efficiency was validated using the FACS protocol described in the methods section.

### Viruses and infection

HCMV ΔUS14-22 used in this paper is HCMV TB40/e-GFP ΔUS14-22, in which IRS1 and UL1 to UL6 are missing due to the insertion of the BAC vector, containing a GFP reporter under the CMV promoter, and deleted for US14-22 (deletion positions 211,755-220,056). The virus was prepared as previously described^39^.

HCMV TB40/e_GFP deletion mutants were generated by BAC recombineering in *E. coli* GS1783^40, 41^ on the same TB40/e-GFP BAC background as the TB40/e-GFPΔUS14-22. For the TB40/e-GFPΔUS14-16, TB40/e-GFPΔUS16-18, TB40/e-GFPΔUS17-20 and TB40/e-GFPΔUS19-22 mutants the indicated gene regions were deleted by replacing them with a kanamycin cassette. The kanamycin cassette was amplified by primers each containing 50 bp homology to the TB40/e-GFP BAC sequence. Homology regions of primers were flanking the gene regions of interest. The resulting recombination fragment was electroporated into *E. coli* GS1783 using Bio Rad 0.2-cm electrode gap cuvettes and a Bio Rad Gene Pulser (2.5 kV, 25 mF, 200 V). Bacteria were plated on chloramphenicol (25 µg/ml)/kanamycin (20 µg/ml) selection plates. After 24 hours colonies were picked and checked for kanamycin cassette integration by colony PCR. The correct knock-in of the kanamycin cassette was verified by Sanger sequencing. TB40/e-GFP-US17stop, TB40/e-GFP-US18stop, TB40/e-GFP-US19stop and TB40/e-US20stop mutants were generated by replacing the first start codon (ATG) of each gene of interest with a stop codon (TAA). First, a rpsL-kana cassette was amplified and electroporated into *E. coli* GS1783 as described above. Homology regions of the primers were flanking the start codon of the genes of interest. Bacteria were selected on kanamycin (20 µg/ml) selection plates and insertion of the cassette was checked by colony PCR. For a second recombineering step the recombination fragment was generated by complementing 80 bp sense and antisense primers. Within these primers the 3 bp stop codon was flanked by 30–40 bp regions homologous to the TB40/e-GFP BAC sequence in the region where the rpsL-kana cassette is then replaced by the stop codon TAA. After electroporation bacteria was plated on streptomycin (5 mg/ml)/kanamycin (20 µg/ml) selection plates. Clones resistant to streptomycin were picked and checked for rpsL-kana cassette removal by colony PCR. The correct replacement of the start codon to the stop codon was verified by Sanger sequencing. The virus was prepared as previously described^42^. Primers used for the generation of the viruses are described in supplemental Tables S2 and S3.

HCMV TRUS20HA and TRΔUS20 were generated and produced as previously described^33^.

Briefly, HFF cells were used to propagate viral stocks. Stocks were purified and concentrated using ultra-centrifugation. Viral stocks were tittered on HFF cells using the plaque assay method and aliquots of viral stocks were preserved in −80 °C until the day of infection. Infections were performed as previously described^31^. All infections for FACS and confocal microscopy experiments were performed with MOIs of 0.1–1, and for FACS presented results are from the infected-cells gate only. All other experiments were performed with MOI ≥ 1.

### Flow cytometry

Cells were split and grown in equal densities for at least 24 hours prior to resuspension or infection. Resuspended cells were counted and equal numbers of cells were stained for 1 hour on ice using primary antibodies (0.25ug/well) diluted in FACS medium (1x PBS, 0.05% Bovine Serum Albumin, 0.05% NaN_3_). Afterwards cells were washed with FACS medium and when needed stained for 30 minutes on ice with secondary antibodies (0.75 ug/well) diluted in FACS medium. Stained cells were read using a FACSCalibur machine (BD Bioscience). In all experiments in which an infection with a GFP^+^ virus is described, the results are of gated GFP^+^ cells.

### Quantitative real-time PCR

Cells were split and grown in equal densities for at least 24 hours prior to lysis. Cells were lysed and RNA was isolated using the Quick RNA Miniprep kit (Zymo). cDNA was prepared from 0.25–1 μg total RNA using mMLV reverse-transcriptase (Invitrogen) and oligoDT primers. The cDNA was mixed with 3 μM of each of the primers (forward and reverse) and 5 μl of Platinum SYBR Green qPCR SuperMix-UDG w/ROX (Invitrogen) was added. B7-H6 was compared between samples, HCMV US9 was measured as a control for infection levels, and hUBC together with hHPRT were used as endogenous reference genes. The quantitative real-time PCR reaction took place on a QuantStudio12K Flex Real Time PCR System (Applied Biosystems).

### Western blotting

Cells were split and grown in equal densities for 72 hours prior to lysis. In the relevant experiments the cell culture growth media was replaced with a fresh media or fresh media containing 10 μM Epoxomycin, 500 μM Bortezomib, 50 nM Concanamycin A, 100 μM Leupeptin or 1:100 (vol./vol.) DMSO ten hours before lysis and harvesting. Lysis was performed in lysis buffer (0.6% SDS, 10 mM TRIS (pH 7.4), and 1 mM PMSF together with Aprotinin (Sigma) as protease inhibitors) and lysates were frozen in −80 °C for at least 24 hours. Lysates were mixed with loading dye containing 2-mercaptoethanol and separated on an acrilamide gel. Next, proteins were transferred to a nitrocellulose membrane, the membrane was blocked for one hour in room temperature (1x PBS, 5% skim milk, 0.4% Tween) and stained using the primary and secondary antibodies described earlier. Membranes were developed using the EZ-ECL kit (Biological Industries) and analyzed using the Image Lab software (Bio-Rad).

### Immunofluorescent staining and confocal microscopy

Cells were plated and grown on 8-chamber glass slides, fixed and permeabilized for 10 minutes by −20 °C methanol, and blocked overnight in 4 °C using CAS-block (Thermo Fisher Scientific) supplemented with human serum. The following day the cells were stained with the relevant primary antibodies diluted in CAS-block overnight in 4 °C. Afterwards the slides were washed and incubated with fluorescently-labeled secondary antibodies for 2 hours in room temperature. The cells were washed again, stained with 4′,6-diamidino-2-phenylindole (DAPI) for 4.5 minutes in room temperature, and visualized using an Olympus Fluoview FV1000 confocal microscope. Image quantification was performed using the Olympus Fluoview FV1000 software.

### NK cell cytotoxicity assays

Natural killer cells cytotoxicity experiments were performed as previously described^31^.

### Statistical methods

A Student’s t-test or an ANOVA test were performed in order to check for significance as stated in the text. Significance was considered when p < 0.05 or lower, as stated in the text. For correlation measurement Pearson’s coefficient was calculated.

All experiments and experimental protocols described in this paper were performed in accordance with the Hebrew University safety and ethics guidelines and regulations.

## Electronic supplementary material

Supplemental information
