# Supplementary material for: Human cytomegalovirus escapes immune recognition by NK cells through the downregulation of B7-H6 by the viral genes US18 and US20
Source: Sci Rep. 2017 Aug 17;7:8661. doi: 10.1038/s41598-017-08866-2 (PMC5561058; doi:10.1038/s41598-017-08866-2)
Supplement: Supplementary file 1 — Supplemental information [file 41598_2017_8866_MOESM1_ESM.pdf]

Supplemental Information

Human cytomegalovirus escapes immune recognition by NK cells through the downregulation of B7-H6 by the viral genes US18 and US20

Yoav Charpak-Amikam, Tobias Kubsch, Einat Seidel, Esther Oiknine-Djian, Noemi Cavaletto, Rachel Yamin, Dominik Schmiedel, Dana Wolf, Giorgio Gribaudo, Martin Messerle, Luka Cicin-Sain, and Ofer Mandelboim

Supplemental figures

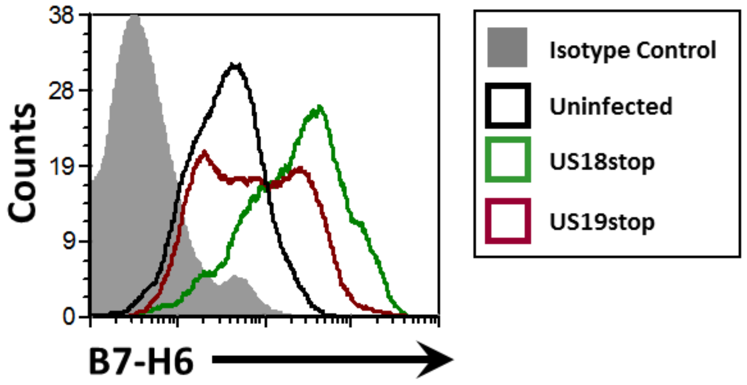

Supplemental Figure S1 – HCMV US18stop fails to downregulate exogenously expressed FLAG-tagged B7-H6. FACS staining of B7-H6 surface expression in ARPE19 cells overexpressing B7-H6-FLAG and infected with different HCMV strains. The filled gray histogram represents staining with an isotype control antibody. The black empty histogram represents uninfected cells. The green empty histogram represents cells infected with TB40/e US18stop. The red empty histogram represents cells infected with TB40/e HCMV US19stop. No significant differences were observed between isotype control stainings of each infection, so a representative isotype control staining derived from the uninfected cells sample is presented.

Supplemental Tables

#	Name	Sequence	Description
1	US17-His F	CCGCGGCCGCGCCGCCACCATGTCTCCGAACTCAGAGGCC	Forward primer used for amplification and cloning of HCMV US17-His
2	US17-His R	GGGATCCTTAATGATGATGATGATGATGCGCCATGGTTCGCGTGAG	Reverse primer used for amplification and cloning of HCMV US17-His
3	US18-His F	AAGCGGCCGCGCCGCCACCATGGGCGACACCGC	Forward primer used for amplification and cloning of HCMV US18-His
4	US18-His R	AACTCGAGTTAATGATGATGATGATGATGCAACAAGCTGAGGAGACTCAC	Reverse primer used for amplification and cloning of HCMV US18-His
5	US19-His F	CCGCGGCCGCGCCGCCACCATGCTTCATGTCGTCCCGCTA	Forward primer used for amplification and cloning of HCMV US19-His
6	US19-His R	GGGATCCTCAATGATGATGATGATGATGTGGGCTCCACAACCAGAGC	Reverse primer used for amplification and cloning of HCMV US19-His
7	US20-His F	AAGCGGCCGCGCCGCCACCATGCAGGCGCAGGAG	Forward primer used for amplification and cloning of HCMV US20-His
8	US20-His R	AACTCGAGTTAATGATGATGATGATGATGGACTTCCCCGTCGTACTGG	Reverse primer used for amplification and cloning of HCMV US20-His
9	US20-HA F	AAGGATCCGCCGCCACCATGCAGGCGCAGGAG	Forward primer used for amplification and cloning of HCMV US20-HA
10	US20-HA R	AAAGGTACCTTATACCCATACGATGTTCCAGATTACGCTGGACTTCCCCGTCGTACTGG	Reverse primer used for amplification and cloning of HCMV US20-HA
11	B7-H6-C′Flag F	AAAGGATCCGCCGCCACCATGACGTGGAGGGCTG	Forward primer used for amplification and cloning of human B7-H6
12	B7-H6-C′Flag R	AAATGTACATTACTTGTCGTCATCGTCTTTGTAGTCCTGTAGGGGTAACAGTAAAGTTG	Reverse primer used for amplification and cloning of human B7-H6
13	B7-H6 F	TCACCAAGAGGCATTCCGAC	Forward primer used to quantify B7-H6 in quantitative real-time PCR experiments
14	B7-H6 R	TGGGGAAGCCACAACTTCAA	Reverse primer used to quantify B7-H6 in quantitative real-time PCR experiments
15	US9 F	AACGCCCTCAGACTTGGAAC	Forward primer used to quantify US9 in quantitative real-time PCR experiments
16	US9 R	CTACCTGGACACCGAAGCTG	Reverse primer used to quantify US9 in quantitative real-time PCR experiments
17	hUBC F	ATTTGGGTCGCGGTTCTTG	Forward primer used to quantify hUBC in quantitative real-time PCR experiments
18	hUBC R	TGCCTTGACATTCTCGATGGT	Reverse primer used to quantify hUBC in quantitative real-time PCR experiments
19	hHPRT F	TGACACTGGCAAAACAATGCA	Forward primer used to quantify hHPRT in quantitative real-time PCR experiments
20	hHPRT R	GGTCCTTTTCACCAGCAAGCT	Reverse primer used to quantify hHPRT in quantitative real-time PCR experiments

Supplemental Table S1 – DNA primers used in the study

#	Name	Sequence	Description
1	US14-16 deletion_fw	CCCCACGGATCTCGCGTCTTAGACGCGCGGTCATATAGCCTCCGGCTGTCcgatttattcaacaaagccac	Forward primer used for deleting the HCMV US14-16 genomic region
2	US14-16 deletion_rv	GAGTGAACGGGTGAGCGTCTCGGTGGAGTCTTCTTATAAACCAGCGGGTCttagaaaactcatcgagcat	Reverse primer used for deleting the HCMV US14-16 genomic region
3	US16-18 deletion_fw	ACTGTTTCATCGACGCCTACCTTAGACCGACAGCGGTCGTAAGCGGCAGCcgatttattcaacaaagccac	Forward primer used for deleting the HCMV US16-18 genomic region
4	US16-18 deletion_rv	CGTTCTCTGGAAACGGCTGCTCTGTCCGAAAACCAGTTCCGAACGAAAATttagaaaactcatcgagcat	Reverse primer used for deleting the HCMV US16-18 genomic region
5	US17-20 deletion_fw	AGAGAAGGGTAGGTGCGCCGCAGCGGCTTTGTGCCGAGACCGTCGCCACCcgatttattcaacaaagccac	Forward primer used for deleting the HCMV US17-20 genomic region
6	US17-20 deletion_rv	TTGGTGGAGACGGCCGGCGCGGCGGGTGGGGGAAACGACGAGTTTTTCCGttagaaaaactcatcgagcat	Reverse primer used for deleting the HCMV US17-20 genomic region
7	US19-22 deletion_fw	TTTCGCGCAGCGCGCTTTATCCGATTCGCTGTCGAGACGGCTTTGCCGGCcgatttattcaacaaagccac	Forward primer used for deleting the HCMV US19-22 genomic region
8	US19-22 deletion_rv	ACGTCACGAGTGTGGTCAAACCGTGGCGGCACCCTGTATCCGACCCGTCGttagaaaactcatcgagcat	Reverse primer used for deleting the HCMV US19-22 genomic region

Supplemental Table S2 – DNA primers used for the generation of HCMV block deletion mutants

#	Name	Sequence	Description
1	US17up-Kan_fw	ACACTCTATAAACGGTTTCTCATACGCGCCTTTTGATAGCCACCGCCGTCCCGGAATTGC CAGCTGGGGCGCCC	Forward primer used in the cloning of the kanamycin cassette into US17
2	US17down-Kan_rv	CGCGGGGGTGGGGGCGCCCAGGCCGTCCCGGTGGCCTCTGAGTTCGGAGAGGCCGCT CTAGAACTAGTGGATCC	Reverse primer used in the cloning of the kanamycin cassette into US17
3	US17STOPinsrt_f	CTCATACGCGCCTTTTGATAGCCACCGCCGTCTAATCTCCGAACTCAGAGGCCACCGGG ACGGCCTGGGCGCCCCCACCC	Forward primer used in the insertion of stop codon into US17
4	US17STOPinsrt_r	GGGTGGGGGCGCCCAGGCCGTCCCGGTGGCCTCTGAGTTCGGAGATTAGACGGCGGT GGCTATCAAAAGGCGCGTATGAG	Reverse primer used in the insertion of stop codon into US17
5	US18up-Kann_fw	ACTGTTTCATCGACGCCTACCTTAGACCGACAGCGGTCGTAAGCGGCAGCCCGGAATT GCCAGCTGGGGCGCCC	Forward primer used in the cloning of the kanamycin cassette into US18
6	US18down-Kan_rv	ATGGTGACCGTCGGCGACTCATGGTGTTCGGAAACCGAGGCGGTGTCGCCGGCCGCTC TAGAACTAGTGGATCC	Reverse primer used in the cloning of the kanamycin cassette into US18
7	US18STOPinsrt_f	GACGCCTACCTTAGACCGACAGCGGTCGTAAGCGGCAGCTAAGGCGACACCGCCTC GGTTTCCGAACACCATGAGTCGCC	Forward primer used in the insertion of stop codon into US18
8	US18STOPinsrt_r	GGCGACTCATGGTGTTCGGAAACCGAGGCGGTGTCGCCTTAGCTGCCGCTTACGAC CGCTGTCGGTCTAAGGTAGGCGTC	Reverse primer used in the insertion of stop codon into US18
9	US19down-Kan_rv	AGGTAGGGGACTACCTCCTCGACGGTCCATTCTAGCGGGACGACATGAAGGGCCG CTCTAGAACTAGTGGATCC	Forward primer used in the cloning of the kanamycin cassette into US19
10	US19up-Kan_fw	CTAATGCCTATAAAACCGCGCCCGTTTTCACAGCAGCCGCGCTTGTCGCCCCGGAAT TGCCAGCTGGGGCGCCC	Reverse primer used in the cloning of the kanamycin cassette into US19
11	US19STOPinsrt_f	AAAACCGCGCCCGTTTTCACAGCAGCCGCGCTTGTCGCCTAACTTCATGTCGTCCCG CTAGAATGGACCGTCGAGGAGGTA	Forward primer used in the insertion of stop codon into US19
12	US19STOPinsrt_r	TACCTCCTCGACGGTCCATTCTAGCGGGACGACATGAAGTTAGGCGACAAGCGCGG CTGCTGTGAAAACGGGCGCGGTTTT	Reverse primer used in the insertion of stop codon into US19
13	US20down-Kan_rv	TCGAGAGCCTCCATGCGGGAGAGCAGCAGCGCGTTAGCCTCCTGCGCCTGGGCCG CTCTAGAACTAGTGGATCC	Forward primer used in the cloning of the kanamycin cassette into US20
14	US20up-Kan_fw	AGAGAAGGGTAGGTGCGCCGCAGCGGCTTTGTGCCGAGACCGTCGCCACCCCGGA ATTGCCAGCTGGGGCGCCC	Reverse primer used in the cloning of the kanamycin cassette into US20
15	US20STOPinsrt_f	GGTGCGCCGCAGCGGCTTTGTGCCGAGACCGTCGCCACCTAACAGGCGCAGGAGG CTAACGCGCTGCTGCTCTCCCGCAT	Forward primer used in the insertion of stop codon into US20
16	US20STOPinsrt_r	ATGCGGGAGAGCAGCAGCGCGTTAGCCTCCTGCGCCTGTTAGGTGGCGACGGTCT CGGCACAAAGCCGCTGCGGCGCACC	Reverse primer used in the insertion of stop codon into US20

Supplemental Table S3 – DNA primers used for the generation of HCMV translation stop mutants
